# Transgender-inclusive measures of sex/gender for population surveys: Mixed-methods evaluation and recommendations

**DOI:** 10.1371/journal.pone.0178043

**Published:** 2017-05-25

**Authors:** Greta R. Bauer, Jessica Braimoh, Ayden I. Scheim, Christoffer Dharma

**Affiliations:** Epidemiology and Biostatistics, Schulich School of Medicine & Dentistry, The University of Western Ontario, London, Ontario, Canada; University of Westminster, UNITED KINGDOM

## Abstract

Given that an estimated 0.6% of the U.S. population is transgender (trans) and that large health disparities for this population have been documented, government and research organizations are increasingly expanding measures of sex/gender to be trans inclusive. Options suggested for trans community surveys, such as expansive check-all-that-apply gender identity lists and write-in options that offer maximum flexibility, are generally not appropriate for broad population surveys. These require limited questions and a small number of categories for analysis. Limited evaluation has been undertaken of trans-inclusive population survey measures for sex/gender, including those currently in use. Using an internet survey and follow-up of 311 participants, and cognitive interviews from a maximum-diversity sub-sample (n = 79), we conducted a mixed-methods evaluation of two existing measures: a two-step question developed in the United States and a multidimensional measure developed in Canada. We found very low levels of item missingness, and no indicators of confusion on the part of cisgender (non-trans) participants for both measures. However, a majority of interview participants indicated problems with each question item set. Agreement between the two measures in assessment of gender identity was very high (K = 0.9081), but gender identity was a poor proxy for other dimensions of sex or gender among trans participants. Issues to inform measure development or adaptation that emerged from analysis included dimensions of sex/gender measured, whether non-binary identities were trans, Indigenous and cultural identities, proxy reporting, temporality concerns, and the inability of a single item to provide a valid measure of sex/gender. Based on this evaluation, we recommend that population surveys meant for multi-purpose analysis consider a new Multidimensional Sex/Gender Measure for testing that includes three simple items (one asked only of a small sub-group) to assess gender identity and lived gender, with optional additions. We provide considerations for adaptation of this measure to different contexts.

## Introduction

### The need for trans-inclusive measures of sex/gender

Until recently, developers of population surveys tended to give little thought to methods for ascertaining biological sex and social gender. In 2004, an authoritative guide on developing questionnaire items for telephone surveys stated that sex/gender should be queried with “what is your sex? (male or female)”, if not “obvious” from the respondent’s name or voice. The authors add that “most people will recognize it [asking about sex/gender] as merely a formality” [1]. Yet, survey measures of sex/gender are neither obvious nor mere formalities for transgender (trans) persons, whose gender identities differ from societal expectations for their birth-assigned sex. Trans persons represent an estimated 0.6% of the United States adult population [2] and available data indicate that they face substantial disparities in health and health care access [3]. Knowledge of the sociodemographic characteristics, health needs, and social experiences of trans populations has been constrained by the lack of measures to identify trans persons in population surveys [4–6].

In recent years, government and research organizations have increasingly sought to remedy this exclusion by developing and fielding trans-inclusive sex/gender measures in population surveys and health surveillance systems [7, 2, 8–10]. Trans-inclusive data collection is motivated by concerns for equity and human rights, as well as data quality. Misclassification of trans persons degrades the quality of data on cisgender (non-trans) persons. Moreover, the assumptions embedded in single, binary sex/gender items—e.g., that individuals who indicate being female have cervices—pose threats to validity [11]. At the same time, more sophisticated measurement of sex and gender dimensions offers the opportunity to develop more detailed causal models of the impacts of multiple dimensions of biological sex and social gender on health outcomes for both trans and cisgender persons [4, 12].

### Considerations for trans-inclusive sex/gender measures

In broader population surveys, measures need to be consistently understood by all respondents [13]. Ensuring broad understanding requires attending to the multiple or poorly defined meanings of some terms (e.g., “sex”), as well as the non-equivalence of abstract terms or concepts across languages [13]. Some approaches that have been encouraged to affirm gender diversity, including check-all-that-apply and write-in response options [14], may be more effective in studies conducted within trans communities that have shared vocabularies than in population health surveys. Open-ended questions can create ambiguity for respondents, as well as difficulties in coding variables [15]; check-all-that-apply items are sensitive to primacy effects (selection of first-ordered suitable option) and allow for seemingly contradictory answers. Moreover, both approaches force researchers to decide in which category to place participants for statistical analysis, a decision better (and more respectfully) made by participants themselves.

General principles of questionnaire design and implementation suggest additional issues for sex/gender measures, such as mode of administration, ease of pronunciation (for interviewer-administered surveys), and item ordering effects [15]. Proxy reporting, where a respondent reports information on other household or family members, is common in household and child surveys. This requires proxy reporters to have relevant information [16], which may not be the case regarding the current gender identity of others.

For cisgender respondents to trans-inclusive survey items, comprehension is of primary concern. Approximately 30% of Americans were unfamiliar with the term “transgender” or unsure of its meaning as recently as 2011 [17]. As most participants in population surveys will be cisgender, confusion among a small proportion could generate a group of “trans” participants composed primarily of misclassified cisgender participants [18].

Transgender respondents face different challenges in responding. They must comprehend which dimension of their sex/gender is being queried (e.g., birth-assigned sex, identity, lived gender), make a judgement regarding the ideal response, and then map that response onto the available options or decide to skip. This may be particularly complex if the sex/gender dimension being queried is unclear, or if response options do not accommodate the diversity of sex and gender within trans communities. One-fifth to one-quarter of trans people in Canada and the United States identify beyond the gender binary (e.g., as “non-binary” or “genderqueer”, [19, 20]). Others identify as women or men with a history of transition, and will not endorse a transgender identity [21]. Some people identify with Indigenous or culturally-specific mixed- or multi-gender identities (e.g., two-spirit), which may not acknowledge a distinction between gender and sexuality. Also, the primacy of gender identity is not a universal concept, and it may have little meaning for some who live their gender in traditional cultural ways that may not match their birth-assigned sex, but with spiritual or cultural role at the forefront rather than identity [22]. Adolescents who are gender non-conforming or questioning may also find response options inadequate [23, 24]. Finally, as variation in social experiences and health outcomes based on sex or gender should be expected within the trans population, it is important that survey items can distinguish trans respondents by gender spectrum (e.g., transfeminine or transmasculine, those assigned male or female at birth, respectively).

### Evaluation of trans-inclusive sex/gender measures to date

Both single-item and two-item measures of gender identity have been evaluated, though to date no single-item measure has adequately identified trans participants. No evaluations of dimensions of sex or gender other than gender identity and birth-assigned sex have been conducted. Tate et al. [21] found that using a single item with four identity options–male, female, transgender, and other–produced frequencies for trans respondents only half that produced when cross-classifying separate items for birth-assigned sex and current gender identity (a “two-step measure”). In addition, that single-item measure did not allow for disaggregation of transfeminine and transmasculine trans persons, and introduced greater missingness (frequency of missing responses).

Community-based surveys of transgender persons began employing two-step measurement of assigned sex and gender identity in the late 1990s [25]. While the specific item wording and order of questions vary, organizations including the Center of Excellence in Transgender Health at the University of California San Francisco [14] and the Williams Institute’s Gender Identity in U.S. Surveillance (GenIUSS) Group [24] now recommend such items for clinical data collection, disease surveillance, and population surveys.

A small number of studies in addition to Tate et al.’s study have evaluated self-administered versions of two-step measures. Lombardi and Banik [26] conducted cognitive interviews with 25 cisgender and 25 transgender people in Ohio, United States. Both groups found the “assigned sex at birth” item straightforward. While some cisgender participants found being asked about both sex and gender redundant, no participants reported difficulties in responding. Reisner et al. [23] evaluated the construct validity of a different set of two-step items in a cohort of U.S. adolescents, and a cognitive interview subsample. They also found that neither trans nor cisgender participants had problems comprehending or responding to the questions. The largest group of trans respondents were those who “do not identify as female, male, or transgender”, highlighting the need for inclusion of non-binary response options in data collection with young people. Finally, Reisner et al. [27] included another version of a two-step sex/gender assessment in a survey of over 35,000 users of a sexual networking website for men who have sex with men in Spanish- and Portuguese-speaking countries. Some challenges were encountered with the question set, including respondents endorsing “prefer not to answer” or mutually exclusive responses. Most of the respondents who wrote in their own gender identity provided a sexual orientation label, reflecting the closer ties between gender and sexuality in Latin American contexts, and underscoring the need for cognitive testing of sex/gender measures among people from diverse cultural backgrounds—including Indigenous people in the Canadian context.

Comparisons across studies are complicated by variations in the measures evaluated. An important limitation of the studies by Tate et al. [21] and Lombardi et al. [26] is that both used versions of the two-step method that include “intersex” or “unknown” options for sex assigned at birth. This is problematic because all infants in the U.S. are assigned either a male or female sex, including those with intersex variations, and this option precludes categorization of respondents by transgender status and gender spectrum [24]. In addition, each study offered a different set of response options for current gender identity. Finally, studies differed in the ordering of sex and gender measures, and no study directly evaluated ordering effects.

Some studies have added a survey item to identify transgender respondents to their usual sex/gender measure. The U.S. Behavioral Risk Factor Surveillance System survey assessed sex/gender indirectly (by voice) and included an opt-in module which asked “Do you consider yourself to be transgender?”. This measure was used to produce the first national estimates of transgender population prevalence for the United States, but does not allow for differentiation by gender spectrum and may underestimate prevalence through misclassification of trans persons who do not identify themselves as “transgender”. Indeed, cognitive testing indicated that while the question was easily understood and answered by cisgender participants, some respondents of transgender experience answered “no” [25]. In an attempt to identify their gender spectrum, a follow-up question was added for those who indicated they were transgender, asking whether they identify as “male-to-female, female-to- male, or gender non-conforming” [2]. Similarly, the Ontario Health Study in Canada added a question “Do you consider yourself to be trans (transgender, transsexual, or a person with a history of transitioning sex?” [28]. Those responding “yes” or “don’t know” were asked follow-up questions on multiple dimensions of sex and gender. This set was designed by Bauer [4] to assess sex and gender dimensions relevant to the multiple uses of the cohort study data, and while in use has not been formally evaluated.

Importantly, sex/gender measures differ regarding dimensions of sex and gender captured. It is generally not clear which dimensions are captured by single items. Currently used “two-step” measures capture three dimensions of sex/gender: sex assigned at birth, current gender identity, and trans status (through cross-classification). Bauer’s [4] multidimensional measure captures these dimensions, plus lived gender, and hormonal and surgical status. Additional dimensions of sex and gender not captured in existing item sets include chromosomal sex, secondary sex characteristics, and conventional masculinity or femininity [4]. Depending on the research questions, different dimensions may be salient. For example, data on hormonal milieu are required in many studies of hormone-linked cancers, while lived gender is relevant to understanding experiences of social stigma amongst trans persons.

Additionally, except for one study [27], trans-inclusive sex/gender measures have been evaluated in predominantly white, young, college-educated, native-born United States samples [26, 23, 21]. Moreover, while developed in colonial countries, none have considered Indigenous conceptualizations of gender. Thus, important questions remain regarding how these measures will perform in a population that is diverse with respect to Indigenous identity, race, ethnicity, religion, immigration history, and education.

### Objectives

This analysis aims to 1) evaluate missingness, comprehension, and clarity for two questionnaire item sets for trans-inclusive sex/gender measures that are currently in use, 2) evaluate agreement between the two measures for the dimension of gender identity, 3) examine the extent to which those coded as trans on gender identity measures may represent other study populations of interest (e.g. those living in a gender different from their birth-assigned sex, those with “male” or “female” hormonal milieus), 4) explore, using an intersectional approach, how conceptualizations of sex/gender across different ages, ethnicities, and sexualities relate to participants’ approaches to these survey questions, and 5) conduct a mixed-methods analysis of methodological issues in trans-inclusive sex/gender measures for population surveys. Our ultimate goal is to propose next steps for the development of trans-inclusive sex/gender items that will function well in a multi-cultural, multi-generational Canada, and to lay out considerations in adapting these to other contexts.

## Materials and methods

### Recruitment strategy, data collection and study samples

Survey data were collected between October 2015 and March 2016 using an online questionnaire. After first reading the letter of information, participants indicated their consent to participate by clicking on a button. Anyone who was 14 years old or older, lived in Canada, and was able to complete an English-language survey was eligible to participate. Consent from a parent or guardian was not required for minors. Methods for this study were approved by the Research Ethics Board at The University of Western Ontario. The study was promoted through Facebook ads, Facebook postings, and e-mails to listservs chosen to generate a diverse sample with high frequencies of sexual and gender minorities; 588 individuals completed the first of two randomly assigned sets of survey items (approximately 5–10 minutes) and provided contact information for follow-up. These participants were contacted via e-mail one to three weeks after participation, with an invitation to either complete the remaining measures (3 minutes) or participate in both the follow-up survey and an immediate semi-structured cognitive interview. Interview participants received a $50 gift card as an honorarium; no honorarium was provided for survey participation.

Interview participants were selected to maximize demographic variation with regard to transgender status, sexual orientation, age, province of residence, immigration status, linguistic background, Indigenous identity, race/ethnicity, education, religiosity, and religious affiliation. Interviews were conducted via telephone or Skype by two of the authors (JB, CD) and were audio recorded; they ranged in length from 24 to 81 minutes, and covered measurement of race/ethnicity and sexual orientation as well as sex/gender.

This mixed-methods analysis is based on participants who completed both surveys (n = 311) and the subgroup who participated in individual interviews (n = 79).

### Trans-inclusive measures of sex/gender

Survey measures evaluated in this study included a measure of multiple dimensions of sex and gender developed by one of the authors [4], originally for the Ontario Health Study, and a two-step question set identified as promising and recommended for further testing by The GenIUSS Group in the U.S. [24]. Both item sets are currently in use in large studies, though there are many other versions of trans-inclusive sex/gender measures also in use. Neither measure has been validated or undergone formal cognitive testing, though the two-step item set is quite similar to other two-step measures that have been studied in select populations. These two item sets were chosen as 1) they are quite different from each other, 2) both were expected to perform better than any single-item question based on prior evaluation and knowledge of both survey methodology and gender identity, and 3) conducting cognitive interviews on two sets that use different wordings and assess different dimensions of sex/gender has the potential to generate rich data on contemporary understanding of sex and gender and to critically inform survey item development.

#### Multidimensional sex/gender items and coding

**Fig 1:**
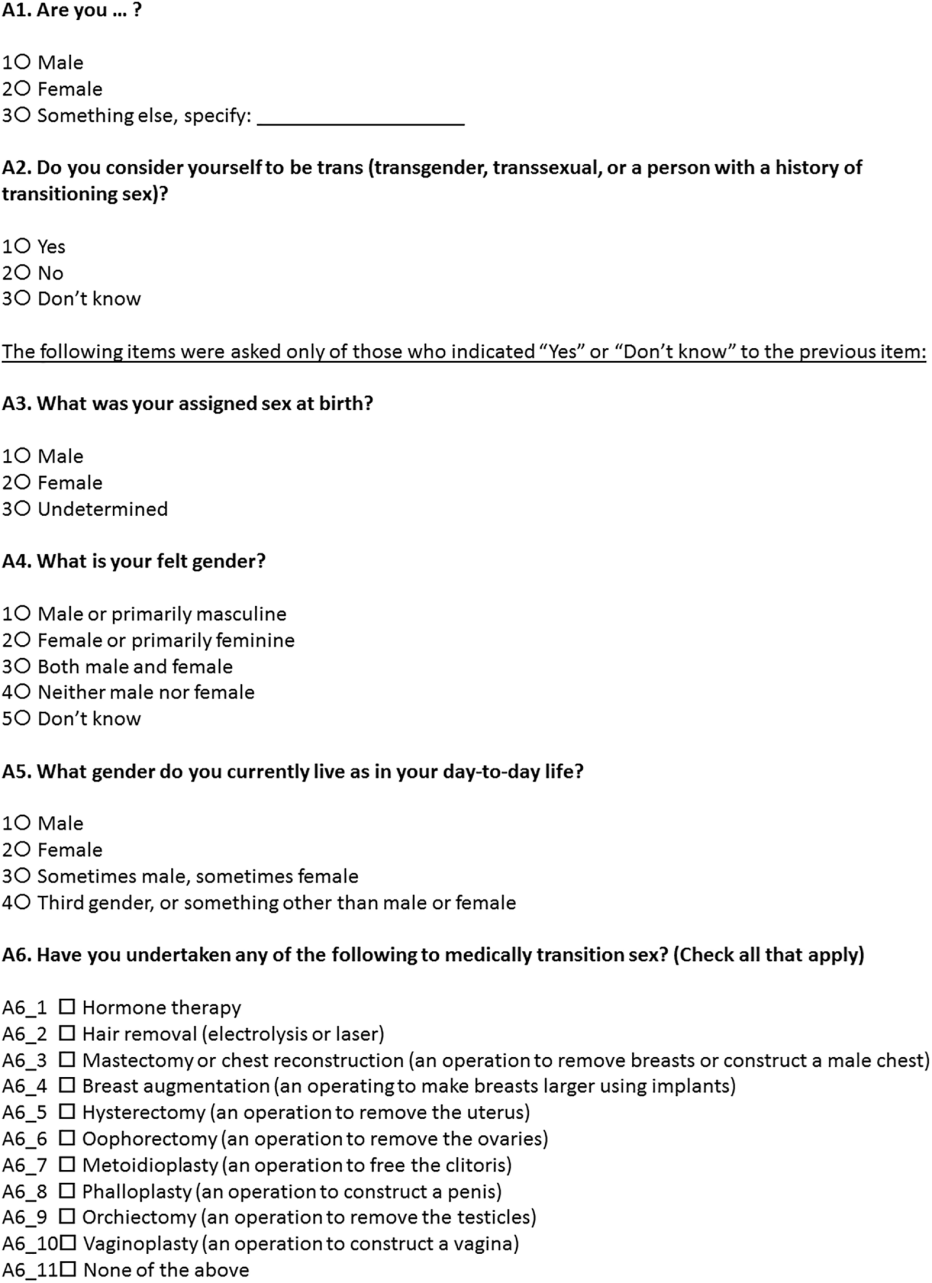
Multidimensional test measure [4].

This measure included two base questions viewed simultaneously and asked of all participants, followed by four additional questions asked only of the sub-group that indicated they were trans or were unsure if they were trans. The measure is included in Fig 1. The two base questions assess gender and trans status, and the four questions for the trans or unsure sub-group assess sex assigned at birth, gender identity, lived gender, and changes to sex (e.g. hormones, surgeries). Fill-in options for “something else” on the first gender item were recoded if necessary (e.g. where a write-in matched a listed option). Items asked only of the trans or unsure sub-group were forward-filled for cisgender participants, to code them as having a consistently male or female sex assigned at birth, gender identity, lived gender, and lack of medical transition. Based on cross-classifying sex assigned at birth with another dimension of gender or sex, five variables were then coded: two versions of gender identity, two versions of lived gender, and one variable for hormonal sex (though genital sex could have been coded as well, if relevant to a health-related research question). For both gender identity and lived gender, two variables were coded, one consisting of all available cross-stratified categories (e.g. assigned male, identified as female or feminine; assigned female, identified as both/neither male or female) and one consisting of four categories: cisgender men, cisgender women, transfeminine persons, and transmasculine persons. SAS code for recoding of variables into categories is included in the supplemental materials (S1 File).

#### Two-step sex/gender items and coding

**Fig 2:**
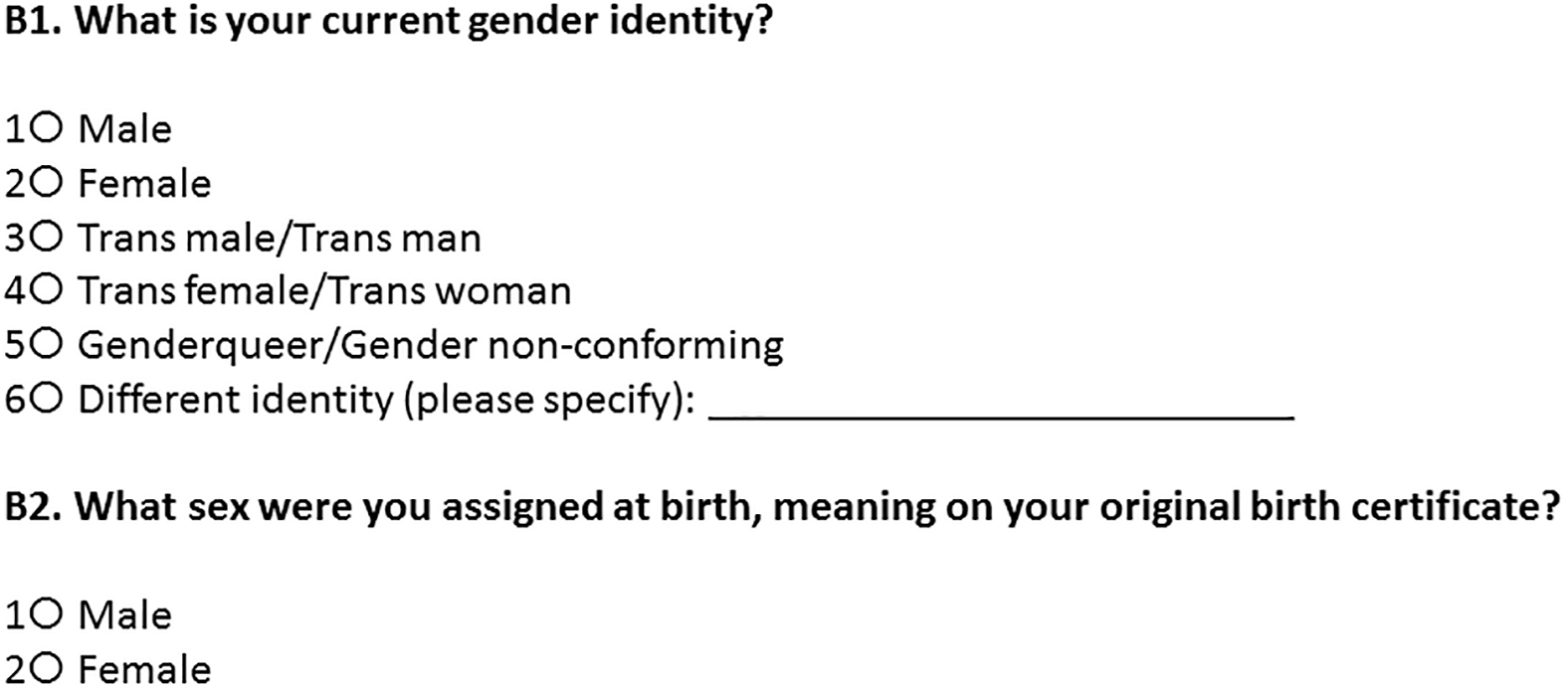
Two-step gender identity test measure [24].

This measure included two items, assessing current gender identity and sex assigned at birth. Item wordings are included in Fig 2. Since the gender identity item offered a fill-in-the-blank option, these items were reviewed and recoded as necessary (e.g. “trans woman” to trans female, “femme” in someone assigned female at birth to female, “agender” to non-binary). Similar to the multidimensional measure of gender identity, two recoded variables were created, one with six categories and one with four. Sample SAS code for recoding is included in the supplemental materials (S2 File).

### Demographic measures

Age and first language of participants were self-reported. Province or territory of residence was determined from the first digit of postal code. Questions on race/ethnicity, Indigenous identity, sexual orientation and education were taken from Canadian Community Health Survey questionnaires [29]. Immigration history was coded from a series of questions asking about personal and parental immigration histories; first generation Canadians were defined as those born in Canada to parents who were both born outside of Canada. Religiosity and religion were assessed using two separate questions, where religion options included non-religious categories such as “atheist” and “agnostic”.

### Interview questions

Survey methodologists have proposed four cognitive steps involved in responding to questionnaire items: comprehension, retrieval, judgement, and reporting [16]. Cognitive interviews are designed to assess participants’ experiences of completing survey items by examining how their comprehension and interpretation of questions and response options orientates a decision or judgement [30, 31]. Cognitive interview participants were asked questions regarding the second questionnaire item set they completed immediately prior to the interview; 43 interviews focused on the multidimensional measure and 36 on the two-step question set. We developed a set of standardized semi-structured interview questions to query how participants understand and respond to the survey questions, beginning with “How did you decide to answer the question?”. This was probed by follow up questions such as “Why did you decide to answer don’t know?” or “Did you think to answer in any other way?”. This was followed by questions that asked how participants conceptualized sex and gender, such as “In surveys often we see the terms, ‘sex’ and ‘gender’. What do these mean to you?”. We then used participants’ conceptualizations of sex and gender (either as equivalent or separate and distinct) to ask questions about how these understandings mapped onto participants’ own experience, such as “How do you understand your own sex and/or gender?”, “Which part of yourself do you consider to be your sex and/or to be your gender?”. As part of an intersectional approach to understanding sex and gender, and to evaluate how measures may perform differently for those at different social intersections, participants were asked to reflect on how other aspects of their identity or experience (e.g. cultural background, sexual orientation) may affect how they understand or present their gender. Finally, interviewees were asked “If surveys could ask about sex and/or gender in ways that would make most sense to you, how should they ask about it?”

The Questionnaire Appraisal System (QAS) was used to assess the two sex/gender measures evaluated [32] for problem types potentially relevant to our items. The QAS was used to identify seven categories of problems: instructions, clarity, assumptions, knowledge or memory, sensitivity or bias, response options, and other problems; categories were not mutually exclusive, so a single problem could be classified under multiple categories. Specific types of problems within each category (16 sub-types) were queried where relevant. These were coded by the interviewers based on issues that emerged in interviews, or asked directly to participants at the end of the interview (where issues were not brought up). Because interviews probed deeply on questions, these reflect problems that may not have been apparent to participants initially in completing the survey.

### Data analysis

Statistical analyses were performed using SAS software version 9.4 [33] to estimate frequencies for demographic and sex/gender measures. To assess dimensions of sex and gender captured by the two-step question, this measure was cross-tabulated with results coded from the multidimensional measure. Interview transcripts were coded using NVivo software version 11 [34]. We used a modified grounded theory approach to structure our analysis, using early transcripts to guide our initial codebook, and modifying it based on themes emerging from interviews as they were analysed. Intersectionality theory [35–37] informed our analysis strategy, in that we paid close attention to the ways that participants’ identities and social positions informed their understanding and experiences of sex and gender, regardless of whether participants themselves framed these issues in an intersectional way [38]. Mixed-methods analysis was conducted as a concurrent triangulation design with an iterative process beginning with quantitative assessment of the QAS items on assessment of problems with survey items, and the analysis of agreement between measures [39].

Given that a majority of interview participants reported problems with survey questions, and that many of the issues emerging in the qualitative data applied to both sets of questions, we present results in a combined Results and Discussion section, focused around issues that emerged in the analysis, rather than a sequential evaluation of each question set.

## Results and discussion

### 1. Sample composition

For both the survey sample and the interview sample, sociodemographic frequencies are presented in Table 1 and sex/gender frequencies in Table 2. Our convenience sample of 311 participants included high proportions of those: assigned female at birth (77.0%), with a postsecondary degree or diploma (77.5%), non-Indigenous white (76.0%), English first language (85.9%) and non-religious (64.3%). Participants were distributed across all age groups, but with only a small number (3) in the 65 and older category; participants were from all provinces except the smallest, Prince Edward Island, but none were from the northern territories. Because sampling strategies aimed to over-recruit sexual and gender minorities, 45.3% of the sample identified as homosexual or bisexual. Demographic categories were more evenly distributed among the interview sample participants (n = 79), as we aimed for a maximum diversity sample to evaluate cognition and measure performance across population sub-groups.

**Table 1:** Sample sociodemographic characteristics, Canadians age 14 and over.

	Survey sample		Interview sample	
	N = 311		N = 79	
	n	%	n	%
**Age**				
14–18	17	5.5	7	8.9
19–24	50	16.1	15	19.0
25–34	113	36.3	21	26.6
35–44	60	19.3	15	19.0
45–54	35	11.3	7	8.9
55–64	33	10.6	13	16.5
65+	3	1.0	1	1.3
**Sex (assigned at birth)**				
Female	237	77.0	51	64.6
Male	72	23.0	28	35.4
**Province or territory**				
Northwest Territories, Nunavut, or Yukon	0	0	0	0
Alberta	20	6.4	8	10.1
British Columbia	57	18.3	21	26.6
Manitoba	7	2.3	4	5.1
New Brunswick	16	5.1	6	7.6
Newfoundland and Labrador	2	0.6	1	1.3
Nova Scotia	14	4.5	4	5.1
Ontario	173	55.6	25	31.7
Prince Edward Islands (PEI)	0	0	0	0
Quebec	11	3.5	7	8.9
Saskatchewan	11	3.5	3	3.8
**Education**				
Less than high school	9	2.9	4	5.1
High school diploma	14	4.5	7	8.9
Some post-secondary	47	15.1	15	19.0
Post-secondary degree or diploma	241	77.5	53	67.1
**Race / ethnicity**				
Indigenous	11	3.6	8	10.3
White (non-Indigenous)	235	76.0	37	47.4
Racialized (non-Indigenous)	63	20.5	33	42.3
**Race / ethnicity ^a^**				
White	256	82.3	52	65.8
South Asian	11	3.5	7	8.9
Chinese	19	6.1	8	10.1
Black	11	3.5	8	10.1
Filipino	2	0.6	2	2.5
Latin American	7	2.3	4	5.1
Arab	3	1.0	1	1.3
Southeast Asian	7	2.3	3	3.8
West Asian	2	0.6	0	0
Korean	1	0.3	1	1.3
Japanese	2	0.6	2	2.5
Another group	7	2.3	5	6.3
**Indigenous identity**				
First Nations, Métis, Inuit	11	3.6	8	10.1
Non-Indigenous	294	94.8	68	86.1
Don’t know	5	1.6	2	2.5
**Religion**				
Christian	75	24.1	18	22.8
Muslim	9	2.9	6	7.6
Jewish	16	5.1	4	5.1
Sikh, Hindu, Buddhist, Neo-pagan	23	7.4	8	10.1
Agnostic or atheist	145	46.6	28	35.4
Other	35	11.3	12	15.2
**Religiosity**				
Religious person	35	11.3	10	12.7
Somewhat religious person	75	24.1	20	25.3
Not a religious person	200	64.3	49	62.0
**Immigration history**				
Immigrant	52	16.7	17	21.5
First generation Canadian	47	15.1	20	25.3
Multi-generation Canadian or Indigenous Canadian	212	68.2	42	53.2
**First language**				
English	267	85.9	62	78.5
French	7	2.3	2	2.5
Other	37	11.9	15	19.0
**Sexual Orientation**				
Heterosexual	151	48.6	26	32.9
Homosexual	57	18.3	20	25.3
Bisexual	84	27.0	22	27.9
Don’t know	18	5.8	11	13.9

^a^ Response options were check-all-that-apply, and so will sum to more than 100%

**Table 2:** Sample sex and gender characteristics, Canadians age 14 and over.

	Survey sample		Interview sample	
	N = 311		N = 79	
	n	%	n	%
**Multidimensional Measure–Individual Items**				
**Sex/gender (“Are you …?”)**				
male	61	19.7	23	29.5
female	216	69.7	44	56.4
something else	33	10.7	11	14.1
**Trans (transgender, transsexual or transitioned)**				
Yes	53	17.0	21	26.6
No	251	80.7	55	69.6
Don’t know	7	2.3	3	3.8
**Assigned sex at birth ^a^**				
Female	237	77.0	51	64.6
Male	72	23.0	28	35.4
Undetermined	0	0.0	0	0.0
**Felt gender ^a^**				
Male or primarily masculine	67	21.7	22	28.6
Female or primarily feminine	221	71.5	44	57.1
Both male and female	8	2.6	5	6.5
Neither male nor female	12	3.9	5	6.5
Don’t know	1	0.3	1	1.3
**Lived gender ^a^**				
Male	68	22.0	21	27.3
Female	223	72.2	45	58.4
Sometimes male, sometimes female	5	1.6	4	5.2
Third gender, or other than male or female	13	4.2	7	9.1
**Medical transition ^a^^,^^b^**				
Hormone therapy	36	11.6	13	16.9
Hair removal	17	5.5	7	9.1
Mastectomy or chest reconstruction	12	3.9	3	3.9
Breast augmentation	3	1.0	3	3.9
Hysterectomy	7	2.3	3	3.9
Oophorectomy	5	1.6	2	2.6
Metoidioplasty	2	0.7	1	1.3
Phalloplasty	1	0.3	1	1.3
Orchiectomy	3	1.0	1	1.3
Vaginoplasty	6	1.9	3	3.9
**Multidimensional Measure–Recodes**				
**Cross-coded gender identity–option 1**				
Identify female, AFAB (cisgender female)	201	65.1	36	46.8
Identify female, AMAB (trans female)	20	6.5	8	10.4
Identify male, AMAB (cisgender male)	49	15.9	17	22.1
Identify male, AFAB (trans male)	18	5.8	5	6.5
Identify non-binary, AFAB	18	5.8	9	11.7
Identify non-binary, AMAB	2	0.7	1	1.3
Don’t know, AFAB	1	0.3	1	1.3
Don’t know, AMAB	0	0.0	0	0.0
**Cross-coded gender identity–option 2**				
Cisgender female	201	65.3	36	46.8
Cisgender male	49	15.9	17	22.1
Transfeminine ^c^	22	7.1	9	11.8
Transmasculine ^c^	36	11.7	14	18.4
**Cross-coded lived gender–option 1**				
Live as female, AFAB (cisgender female)	207	67.0	38	49.4
Live as female, AMAB (trans female)	16	5.2	7	9.1
Live as male, AMAB (cisgender male)	51	16.5	17	22.1
Live as male, AFAB (trans male)	17	5.5	4	5.2
Live as non-binary, AFAB	14	4.5	9	11.7
Live as non-binary, AMAB	4	1.3	2	2.6
**Cross-coded lived gender–option 2**				
Cisgender female	207	67.0	38	49.4
Cisgender male	51	16.5	17	22.1
Transfeminine ^c^	20	6.5	9	11.7
Transmasculine ^c^	31	10.0	13	16.9
**Cross-coded hormonal sex**				
Female, AFAB (cisgender female)	220	71.2	45	58.4
Female, AMAB (trans female)	18	5.8	7	9.1
Male, AMAB (cisgender male)	53	17.2	19	24.7
Male, AFAB (trans male)	18	5.8	6	7.8
**Two-step Gender Identity–Individual Items**				
**Sex (assigned at birth)**				
Female	237	77.0	51	64.6
Male	72	23.0	28	35.4
**Current gender identity**				
Male	54	17.4	20	25.3
Female	200	64.5	34	43.0
Trans male / Trans man	12	3.9	2	2.5
Trans female / Trans woman	14	4.5	6	7.6
Genderqueer / Gender non-confirming	29	9.4	17	21.5
Different identity	1	0.3	0	0.0
**Two-step Gender Identity—Recodes**				
**Cross-coded gender identity–option 1**				
Identify female, AFAB (cisgender female)	194	63.0	32	40.5
Identify female, AMAB (trans female)	20	6.5	8	10.1
Identify male, AMAB (cisgender male)	49	15.9	18	22.8
Identify male, AFAB (trans male)	16	5.2	4	5.1
Identify non-binary, AFAB	26	8.4	15	19.0
Identify non-binary, AMAB	3	1.0	2	2.5
**Cross-coded gender identity–option 2**				
Cisgender female	194	63.0	32	40.5
Cisgender male	49	15.9	18	22.8
Transfeminine ^c^	23	7.4	10	12.7
Transmasculine ^c^	43	13.9	19	24.1

AMAB = assigned male at birth

AFAB = assigned female at birth

^a^ Those who responded “no” to the item on whether they were trans were forward filled to have a birth-assigned sex, gender identity, lived gender and medical transition history consistent with their sex/gender.

^b^ Measure is check-all-that-apply. Total will sum to more than 100%.

c Transfeminine includes both those assigned male at birth who now identify (or live, for the lived gender measure) as either female or a non-binary gender; transmasculine correspondingly includes those assigned female at birth who now identify with (or live as) either male or a non-binary gender.

### 2. Missingness and comprehension of items in the multidimensional measure

Missingness was very low; of 311 survey participants, 1 (0.3%) was missing a response for the MSGM. Of the 43 participants interviewed regarding the multidimensional measure, no one stated that they were unfamiliar with the question or response terminology. While participants were able to complete these two items, some dissatisfaction was common. Frequencies of specific problems from the QAS are presented in Table 3, with 60.5% of participants interviewed on the multidimensional measure items reporting a problem. Problem types most commonly reported included assumptions inherent in the questions, sensitivity or bias, and response categories. Frequencies were high as cognitive interview participants were probed on their understanding of the survey items, and asked to reflect deeply on the questions in order to identify any issues. Details on specific problems identified were captured in the interviews which form the basis of the qualitative analysis.

**Table 3:** QAS frequencies for main problem types identified in cognitive interviews.

	Queried on multidimensional measure		Queried on two-Step	
	(n = 43)		(n = 36)	
	n	%	n	%
Overall				
Any problem with survey item set	26/43	60.5	20/36	55.6
Problem with instructions	6/43	14.0	—	—
Conflicting or inaccurate	4/6	66.7	—	—
Problem with clarity	10/43	23.3	5/36	13.9
Technical	10/10	100.0	5/5	100.0
Reference period	3/9	33.3	2/5	40.0
Problem with assumptions	13/43	30.2	5/36	13.9
Inappropriate	11/13	84.6	2/5	40.0
Constancy	7/13	53.9	2/5	40.0
Double-barrelled	0/13	0.0	—	—
Problem with knowledge/memory	3/43	7.0	2/36	5.6
Knowledge	2/3	66.7	1/2	50.0
Attitude	0/3	0.0	2/2	100.0
Problem with sensitivity/bias	13/43	30.2	7/36	19.4
Sensitive content	6/13	46.2	4/7	57.1
Sensitive wording	6/13	46.2	3/7	42.9
Socially acceptable	2/13	15.4	1/7	14.3
Problem with response categories	11/43	25.6	9/36	25.0
Mismatch	3/10	30.0	1/9	11.1
Technical	3/10	30.0	1/9	11.1
Vague	4/10	40.0	1/9	11.1
Overlapping	1/10	10.0	2/9	22.2
Missing	10/11	90.9	7/9	77.8
Other problems	08/42	19.1	10/36	27.8

### 3. Missingness and comprehension of items in the two-step measure

Of 311 survey participants, 2 (0.6%) were missing for the two-step measure. Among 36 interview participants who were queried on this measure, no one stated that they were unfamiliar with the response options included, though some cisgender persons could not specifically define terms used in each gender option. Also similar to the multidimensional measure, dissatisfaction was high in our cognitive interview sample who were asked to reflect in depth on the measure. Frequencies of specific problems from the QAS are presented in Table 3, with 55.6% of participants interviewed on the two-step measure reporting a problem. The most frequently reported problem types were sensitivity or bias, response categories, and other problems.

### 4. Understanding among cisgender participants

Both measures were clear and straightforward for cisgender participants. When asked about his decision-making in the multidimensional measure, one participant said, “I think this question’s pretty obvious. Like, I’m male. I don’t know how else to explain [laughs] it better.” Similarly, another participant explained her ease with the two-step measure as almost automatic: “I think the only thinking that I really did was actually reading the question just to make sure I clicked the right one. [Laughing] […] I know that I’m female, and I’ve always identified as female. And I know what my birth certificate says.”

While these items worked for cisgender participants themselves, those who were lesbian, gay or bisexual more often recognized the limitations of both measures for trans people. This is not to say that straight cisgender participants were unknowledgeable about gender minorities or the changing social milieu organizing notions of sex and gender. For example, when asked about his understanding of the two-step measure, an older participant (cis man, 65+, white, multi-generation Canadian, Western Canada) spoke about the “current fashion” today that he describes has replaced the term “sex” with “gender” and reclaimed the previously taboo term “queer.” Although this revised way of thinking does not mirror his own, he did not report any challenges with the question and was easily able to place himself within the response categories.

Our findings are consistent with other research showing that cisgender participants did not have comprehension difficulties with trans-inclusive survey measures [26, 23]. However, Canadians are well-educated in general, and our sample even more so. Additional testing among those with lower levels of literacy would be helpful.

### 5. Poor performance of a single item–trans participants versus trans identities

The first question in the multidimensional measure (“Are you …? Male, Female, Something else; specify _____”) reflects a question sometimes asked as a single item. While it was clear and easily answered by cisgender participants, it did not identify trans participants, nor did it clearly identify birth-assigned sex or gender identity. Among 23 transfeminine survey participants, 9% selected “male”, 61% “female” and 30% “other. Among 41 transmasculine survey participants, 21% selected “male”, 19% “female” and 60% “other”. As trans participants responded with different dimensions of their own sex/gender, it is important to note that even with the addition of a second question as to whether one was trans, transfeminine and transmasculine participants could not be differentiated.

While this item alone did not perform well, cognitive interview participants did generally find it acceptable, one commenting with a laugh that “[T]here seem to be more questions now that have gone from a gender binary to a gender trinary.” However, this item was cognitively taxing for trans interview participants, who tried to figure out exactly what the researchers were asking, and reached different conclusions. Our results are consistent with previous research showing that single measures under-identify trans respondents who choose “male” or “female” over other options [40, 21]. This highlights the importance of any trans-inclusive sex/gender measure allowing for the identification of trans participants (such as the following) who identify simply as men or women.

I recognize that I have transitioned but it’s not part of my identity. So, I don’t really identify as a trans person […] But I generally do say like I’m a person with a history of like, a medical history whatever, more so. (trans man, 25–34, white, multi-generation Canadian, Ontario)

This distinction between trans identity and trans experience was clear in the gender identity options chosen by those who were ultimately classified on the two-step measure as trans; of 66 participants coded as trans, 26 chose an identity as trans man or trans woman, 11 as man or woman, and 29 as genderqueer or gender non-conforming. Some participants found the separate categories of, for example, “male” and “trans male/trans man” to be problematic, seeing “trans male/trans man” as a lesser type of man. Thus, while a single item may be attractive to researchers in preserving a shorter survey length, even single items that allow for identification of trans gender spectrum cannot identify trans participants who identify solely as men or women.

### 6. Agreement between measures

Both measures captured the dimension of trans-ness based on gender identity, using birth sex cross-stratified with gender identity, and agreement between these two measures was very high (Cohen’s K = 0.9081). Results are presented in Table 4. This indicates that surveys using the multidimensional measure and this version of the two-step measure will capture identity-based trans populations that are substantively similar.

**Table 4:** Agreement between gender identity measures.

		Multidimensional measure: Gender identity						
		Missing	Cis woman	Cis man	Trans man	Non-binary (AFAB)	Trans woman	Non-binary (AMAB)
Two-step measure: Gender identity	Missing	0	1	1	1	0	0	0
	Cis woman	1	193	0	0	0	0	0
	Cis man	0	0	48	1	0	0	0
	Trans man	0	0	0	15	1	0	0
	Non-binary (AFAB)	0	7	0	1	17	0	0
	Trans woman	0	0	0	0	0	20	0
	Non-binary (AMAB)	1	0	0	0	0	0	2

AFAB = assigned female at birth

AMAB = assigned male at birth

Cohen’s K = 0.9081

The only discordant cell with more than one participant included seven participants who were identified as cisgender women on the multidimensional measure and as non-binary (assigned female at birth) on the two-step measure; these accounted for nearly all of the discrepancy in frequency of trans persons by identity (66 trans persons for two-step measure versus 58 by the multidimensional measure). All seven indicated “no” to the multidimensional measure’s item “Are you trans (transgender, transsexual, or a person with a history of transitioning sex)?”, checked the two-step measure’s identity option of “genderqueer/gender non-conforming”, and indicated a sexual minority identity; write-in gender identity responses from this group included “queer” and “female genderqueer questioning”. Of these seven, five were interviewed, including one who indicated that her response was a mistake. The others clearly indicated a female gender presentation, saying they “stick to the norm”, were “never going to pass as anything other than female”, “present myself as female”, and “I’m definitely too female to be androgynous, but there is an androgynous fit-flavour to how I present”.

This discrepancy suggests that the “genderqueer/gender non-conforming” wording in the two-step measure response option may possibly be too broad. We note that the version of the two-step measure we evaluated was one recommended by The GenIUSS Group [24] for further testing, and is less commonly used than a version that does not include this wording.

### 7. Dimensions of sex or gender captured

**Fig 3:**
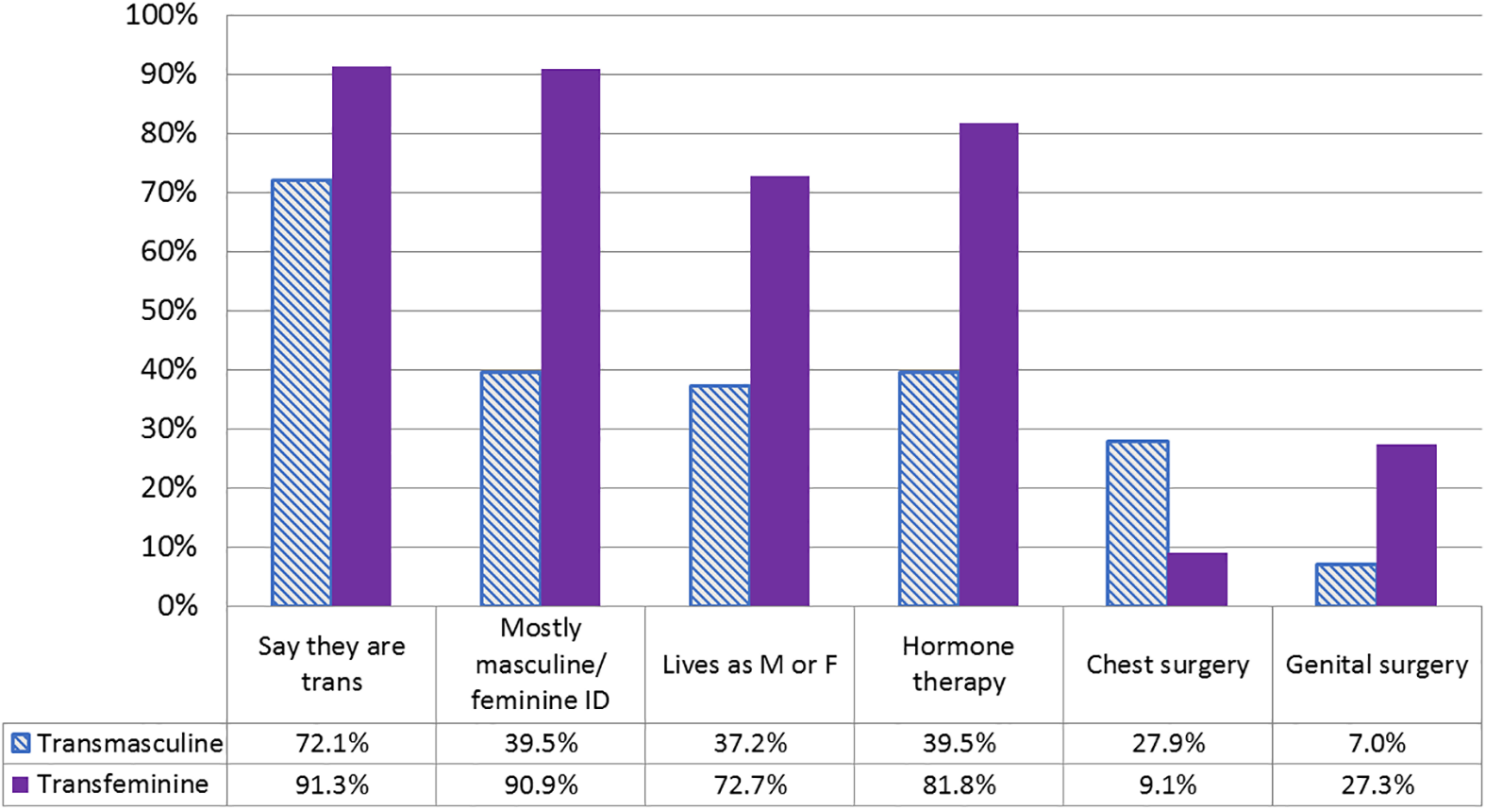
Gender identity as a proxy for other dimension of sex/gender in trans persons.

Among trans participants, gender identity served as a poor proxy for other dimensions of gender or sex that may be of interest to researchers (Fig 3). Of transfeminine spectrum participants, 91.3% identified as trans, 90.9% had a primarily female identity, 72.7% lived as female, 81.8% used hormones, 9.1% had undergone breast surgery, and 27.3% had undergone genital surgery. Proportions for each of these were lower among transmasculine participants, with the exception of chest surgery (27.9%). This is consistent with existing research, showing that trans identity is the most common dimension expressed. Estimates from Ontario show 70% of transfeminine people living in their identified gender (46% full-time) along with 84% of transmasculine people (49% full-time); a smaller proportion were using hormones or had surgeries [20].

Transmasculine and transfeminine gender spectra defined using the two-step measure.

Gender identity and lived gender (often cross-tabulated with sex assigned at birth) will be the dimensions most relevant for population health studies of outcomes shaped by social experiences (e.g. mental health, substance use), and for assessing health-related inequalities on a population level. Collection of both variables also allows for more appropriate grouping of trans and cisgender respondents, based on the dimension most relevant to the outcome under study (which may often be lived, rather than identified gender). In studies with sufficient numbers of trans respondents, gender identity and lived gender can also be cross-tabulated to examine the impacts of identity disclosure or “outness” (see code in the supplemental materials S3 File). However, other studies assess health issues that relate directly to hormonal milieu or to anatomy. Ultimately, the dimensions of sex/gender assessed in a study will need to be considered in light of the purposes for which the data will be used.

### 8. Intersex participants

Our study did not evaluate any measures of sex/gender that were designed to also identify intersex individuals. Intersex refers to those with a range of chromosomal or hormonal conditions that create characteristics that are not sexed consistently male or female; these are sometimes also referred to as disorders of sex development or differences of sex development (DSD). Those who are intersex will typically have a sex assigned at birth, a gender identity, and a lived gender that may be captured with the measures studied. Including intersex in survey items for sex assigned at birth may be problematic in at least two ways; intersex individuals typically are assigned a sex at or shortly after birth and raised in the corresponding gender, and classifying sex at birth as intersex does not allow for the identification of those intersex people who have had trans experience (i.e. has an identity that varies from birth-assigned sex [41]). Intersex participants may be identified through a separate question, for example “Have you been diagnosed with a medically recognized intersex condition?” [42]. Research specific to intersex issues may require more detailed information, as different intersex conditions have different health and social implications.

### 9. Genderqueer, non-binary, bigender and agender persons

Twenty participants reported a non-binary gender identity on the multidimensional measure, and 29 on the two-step measure (including the seven cisgender participants discussed earlier). Write-ins that indicated non-binary identities included “agender”, “gender queer”, “female questioning” and “non-binary”. The multidimensional measure’s gender identity item asked of trans respondents (and those who didn’t know whether they were trans) was interpreted as clear and straightforward for about half of the 14 trans/unsure participants queried on it in interviews, with concerns focused on the ways in which follow up questions may perpetuate a gender binary. For example, one participant reported that the felt gender question was difficult because “it’s hard to distinguish for me between feeling both male and female and feeling neither.”

The issue of whether genderqueer persons are included within a broad definition of “trans” came up in multiple interviews, with genderqueer participants themselves holding differing views on whether they are trans, but agreeing that this was a point of community disagreement. This is illustrated in the following pair of quotes:

But often there’s an assumption that they’re—they’re separate things [trans and non-binary].... I’ve sometimes encountered a little bit of friction maybe around the idea that—I guess I don’t want to be appropriating anyone’s identity either, right. … I actually am non-binary and for me being trans is a pretty broad term, which includes anyone whose gender is different than their assigned sex at birth. And that’s not everyone’s understanding. (genderqueer/trans AFAB, 35–44 years, white/Japanese, immigrant, Western Canada)

So I identify as genderqueer, which I think of as a separate thing from issues of transgender. … Well, [genderqueer] to me means that you conceptualize yourself, I guess primarily not on the strict binary, but of an identity other than the identity that you physically present as, without necessarily—and I guess I draw the line between that and transgender as someone who specifically identifies as a presentation that they may want to physically transform into. … And I understand that that’s not a hundred percent [laughs] how other people see that distinction… (genderqueer/non-trans AMAB, 45–54 years, white, multi-generation Canadian, Atlantic Canada)

For researchers, these differing understandings among genderqueer persons and within broader trans communities highlight the importance of defining the trans population under study, and of not assuming that genderqueer persons will identify as trans.

### 10. Indigenous identities

Some Indigenous participants identified as two-spirit, an umbrella term adopted in 1990 at an intertribal conference in Winnipeg, Canada, as a way of communicating a broad range of traditional Indigenous gender-diverse identities and social roles [43]. Two-spirit represents an assertion of Indigenous identities that may take forms outside of the recent academic formulation of sexual orientation and gender identity as distinct concepts, and outside of identities such as lesbian, gay, bisexual and transgender that have developed within primarily white settler communities. While Indigenous terminology and traditional gender roles may differ between First Nations, they also do not fit neatly into commonly used survey formulations of sexual orientation, as well as gender identity, as they do not make a distinction between the two. One of our participants who reported living in a third gender (or something other than male or female) explained:

Well, I chose gender—gender fluid or genderqueer, because that’s representative of my identity. I do identify as two-spirited, but sometimes that more gender fluid and gender—but having almost kind of like that balance of sometimes that I feel that I have—I’ll have a more of a masculine day and more—as compared to having more of a feminine day. … it fluctuates, and it’s not that I think that I’m transgendered at all. I think whereas some people, they misunderstand that two-spiritedness can be—is considered to be something under the transgender spectrum, when really it’s something that’s more of a spectrum in itself that is inclusive of sexuality and gender identity… but very exclusive to First Nations and Indigenous people. So—and sometimes it’s a lot—a little more complicated than just saying gender fluid [laughs]. (genderqueer/not sure if trans AMAB, 25–34 years, First Nations, Ontario)

As relayed by this participant and other Indigenous participants, two-spirit identities may be more “gender fluid” than “trans”, and more complicated than “gender fluid”, and in fact represent a different paradigm. The distinctions made between two-spirit and other newer gender-diverse identities in North American colonial contexts reflects considerations internationally with regard to the many traditionally held genders. Despite being developed in the U.S. and Canada, neither of the measures we tested had options for a traditional Indigenous identity, and required that those with traditional cultural gender identities categorize their identity as male or female, or under newly emerged terms such as “trans woman” or “genderqueer”. This should be remedied to both provide an appropriate response option and to affirm Indigenous identities in survey measures. We note that this does not preclude also including an Indigenous response option in survey questions on sexual orientation, though this is beyond the scope of this evaluation.

### 11. Medical treatments and medical transition

The multidimensional measure can be adapted to include only information relevant to a particular study’s aims, if needed. While we found no issues with trans participants being unable to answer this question, several trans participants had suggestions. They reported that the stem of the question on hormones and surgeries was embedded within a gender binary (“medical transition”) that assumes that trans people are moving or have moved from one gender to another. As one participant explained, their decision to take hormones was not about medically moving towards an opposite gender. Instead, it was about “help[ing] me feel more congruent with who I am. […]for me it’s more about being androgynous.” the question stem could likely be edited to avoid these implications by changing from “Have you undertaken any of the following to medically transition sex?” to “Have you undertaken any of the following related to your gender?”.

One trans participant also cautioned against researchers making ableist assumptions with regard to what a medical transition does or does not mean with regard to gender.

There are some things that I would never be able to do because they’d be medically contraindicated in my situation. There are other things that—you know, there’s nothing left to do because other kinds of surgeries for other lifesaving interventions, um, didn’t leave anything behind to work with. So the question is kind of fine if you are an otherwise able-bodied person. [trans man, 45–54, white, multi-generation Canadian, Prairies]

This is important in avoiding assumptions that medical procedures represent a more complete transition; indeed research shows that a substantial proportion of trans persons who indicate they have “completed” a medical transition have not had any surgeries [20]. Moreover, this participant’s statement emphasizes that trans (or cis) participants may have had similar surgeries for other medical reasons. We note that if these types of anatomical and hormonal questions are relevant for a research study, researchers should include the relevant items in a broader skip pattern based on sex assigned at birth, to ensure assumptions are not being made regarding the anatomy of *cisgender* participants.

### 12. Order of items

While our survey randomly allocated which of the two measures was given in the initial survey (with the other at follow-up), we did not vary the order of items within each question set. For this online evaluation, the first two items in the multidimensional measure were visible together on the screen, as were both items in the two-step measure. While many cisgender participants did not see much distinction between the two questions (true for both sets), their clarity for trans participants may have been dependent on both being visible. Thus, we recommend that the questions be simultaneously visible for online and paper surveys. We reviewed our interview transcripts for any information to inform ordering of items, and found little evidence for the influence of ordering on decision-making where both were visible. For telephone or in-person interviews in which a participant receives one question at a time, order may matter much more. Thus, we would recommend that interviewer-administered questionnaires begin with the more concrete question, sex assigned at birth. Finally, while we did not evaluate the order of response options within each measure, given the potential for accidental responses, it may be preferable to list more numerically common response options first to avoid error.

### 13. Considerations for self-completed versus proxy-reported surveys

While our findings represent self-reported measures, they also highlight potential concerns with accuracy of proxy-reported measures, for example in censuses or household surveys wherein one member of the household is reporting on all members. Among participants who were classified as trans based on gender identity in the multidimensional measure, 8 of 58 (13.8%) were living in their birth-assigned gender. Moreover, while we did not ask directly about level of disclosure to others in our interviews, the issue did come up. One genderqueer participant reported that “because my family is Muslim it’s easier for me to […] identify [my sex] as female because then it’s, because otherwise it’s problematic.” When asked if they had told their family, they replied “No, no.” While most trans people were aware that they were trans as preadolescents or adolescents [20], there is often a significant time-lag before telling others or beginning to live with a gender presentation consistent with one’s identity. Thus, proxy reporters may not be aware of gender identities that differ from birth sex, but are likely to more accurately report on the lived gender of others. For this reason, assessing the dimension of lived gender may be preferable to gender identity in proxy-reported surveys.

### 14. Temporality

While we were not able to examine changes in sex and gender dimensions over time in our cross-sectional survey, the issue of temporality and changes in identity over the lifecourse emerged among trans (but not cisgender) participants in the interviews. One participant who reports living in a gender other than male or female explains the process of defining their identity.

I initially thought that I was a trans man because I was raised as a girl, and I thought that my only other option, if I wasn’t a girl, meant that I was a boy. … eventually, I kind of realized [..] I’m not a girl, but there has to be more options than this. … So when I found out about being agender, I’m like, yeah, this is it. [agender/trans AFAB, 19–24, white, multi-generation Canadian, Atlantic Canada]

Data collected in cross-sectional surveys must then be understood as a momentary capture of sex/gender dimensions, rather than a capture of stable identity groups or points in a unidirectional process of transition or identity consolidation. Instead, individuals follow a process of reconsideration in light of new knowledge of self, learning of new identity options, undertaking of medical procedures, and/or the social context in which participants were embedded. This poses a special methodological challenge for cohort studies, many of which collect sex/gender only at baseline. Temporal changes indicate the need to also include sex/gender measures at follow-up data collection time-points.

Another participant who lives their gender as sometimes male, sometimes female, discussed temporality not only as an issue in their personal process of adopting identity labels, but with regard to changes in language use more broadly:

So up until maybe the last six or seven months, I never, ever, ever would have identified myself as trans because I did not feel it was a label that I had access to as a non-binary person… there’s even just a lot of discussion within the trans community about what trans means. Like, you’ve got just the word transgender, or you’ve got, like, the trans asterisk [trans*], which apparently people are saying now is oppressive. I don’t know. It’s a very charged word right now. [genderqueer/trans AFAB, 19–24, Caribbean/European, first-generation Canadian, Ontario]

The changes in community language above relay only very recent developments. Another participant with a longer history in trans community explains how language has changed over the decades, and his identity with it:

When I wrote my [newsletter], I was using the word “new men” for trans men, and later “new women” for trans women, ‘cause we were still kind of pretty binary. But then—in the ‘70s and ‘80s—but then it started opening up a bit, and then we used the word, “male transsexuals” or “transman” or “transsexual men”, and vice versa, “transsexual women”. It shifted a bit, and then of course it became much more fluid maybe in the ‘90s and so on. So, my gender evolved a little bit where it became a bit more androgynous. [trans man, 55–64, Indian/white, first-generation Canadian, Ontario]

Repeated cross-sectional population surveys demand stability of measures over time, so that changes in health among sex/gender sub-groups may be accurately documented. Thus, population surveys must rely on language that will not change rapidly with regard to public perceptions of acceptability or understanding of terminology. Given that language is changing rapidly within trans and cultural communities, and may vary regionally and within different age or gender cohorts, it is imperative that language be as simple as possible. This serves the dual function of promoting comparability over time and within population sub-groups, some of whom will be completing surveys in a second or subsequent language, or with cultural understandings from a wide range of Indigenous or immigrant cultural backgrounds.

### 15. Pragmatic considerations

Neither measure evaluated was considered burdensome by participants. However, in the design of population surveys there is always pressure to use the minimum number of items possible, as adding an item often means removing another. Population health surveys are also unique in that they have many secondary data users, and thus it is difficult to select a single dimension of sex/gender that will meet all user needs. Given that single-item measures cannot accurately classify trans participants, cannot clearly differentiate transfeminine and transmasculine participants (without excluding non-binary participants), and are confusing for trans participants, a minimum of two items will be needed. However, given that gender identity is the broadest classification category (since identity often develops early in life and always prior to changes in lived gender or transition-related medical treatment), additional dimensions of sex or gender (where needed) can be identified with minimal burden by asking these questions only of the subset who indicate an identity different from their sex assigned at birth.

Another pragmatic concern involves the use of open-ended or fill-in-the-blank responses as “other, please specify” categories. Participants, including in this evaluation, often enter responses that are substantively similar to included responses. Thus, a reported “other” cannot be understood to be a true “other” response without verification. While an “other” option allows each participant to enter their personal identity, and avoids problems with needing to change the language of response options over time (though not necessarily with changing interpretations), recoding of write-in responses into categories for comparison will be too labour-intensive for many agencies. Write-in responses provided by trans respondents can sometimes be quite idiosyncratic and even impossible to categorize, resulting in data loss. Moreover, we believe it creates an ethical problem in that such questions appear to allow participants to avoid simple categorizations, but then participants’ identities are categorized by researchers after the fact; the final categorization may be inconsistent with a participant’s self-categorization when given those categorical options.

While population surveys are often done confidentially, and sometimes anonymously, the recent increased focus on creating data linkages raises pragmatic concerns regarding consistency or comparability across data sources. Moreover, as governments move toward greater integration of data, some of the same measures being addressed in this paper with regard to population health surveys will apply to collection of sex/gender dimensions in government databases and documents, and to the sharing of this information [44].

### 16. Strengths and limitations of the current study

The use of mixed-methods for this analysis allowed for detailed analysis of two trans-inclusive survey measures, with some issues emerging initially from analysis of quantitative data, and others from the interview data. Our large number of cognitive interviews (n = 79) from a maximum diversity sample, allowed for the inclusion of perspectives from a range of ages (14 to 77), geographic regions (east to west coast), ethnicities, religions, linguistic backgrounds, and immigration histories. The intersectional approach interrogated the relationships between other aspects of identity or social position and sex/gender, to better assess whether and how these measures perform in Canada, a country with a diversity of cultures and religions, and a rapidly changing context for knowledge of trans language and issues that is likely to produce generational effects. While we were able to test agreement between measures using our survey data, the use of a convenience sample precludes applying any of the quantitative results to the Canadian population as a whole. In particular, quantitative results should be interpreted in light of the demographic composition of our sample participants, who were disproportionately female, sexual and/or gender minority, young, university-educated, non-religious, and speakers of English as a first language. A major limitation of this study is that it was conducted in only English. Any national study in Canada will at a minimum be conducted in both official languages, English and French, while studies in the U.S. often include at minimum an option for participation in Spanish.

## Recommended items for further testing: Multidimensional Sex/Gender Measure

**Fig 4:**
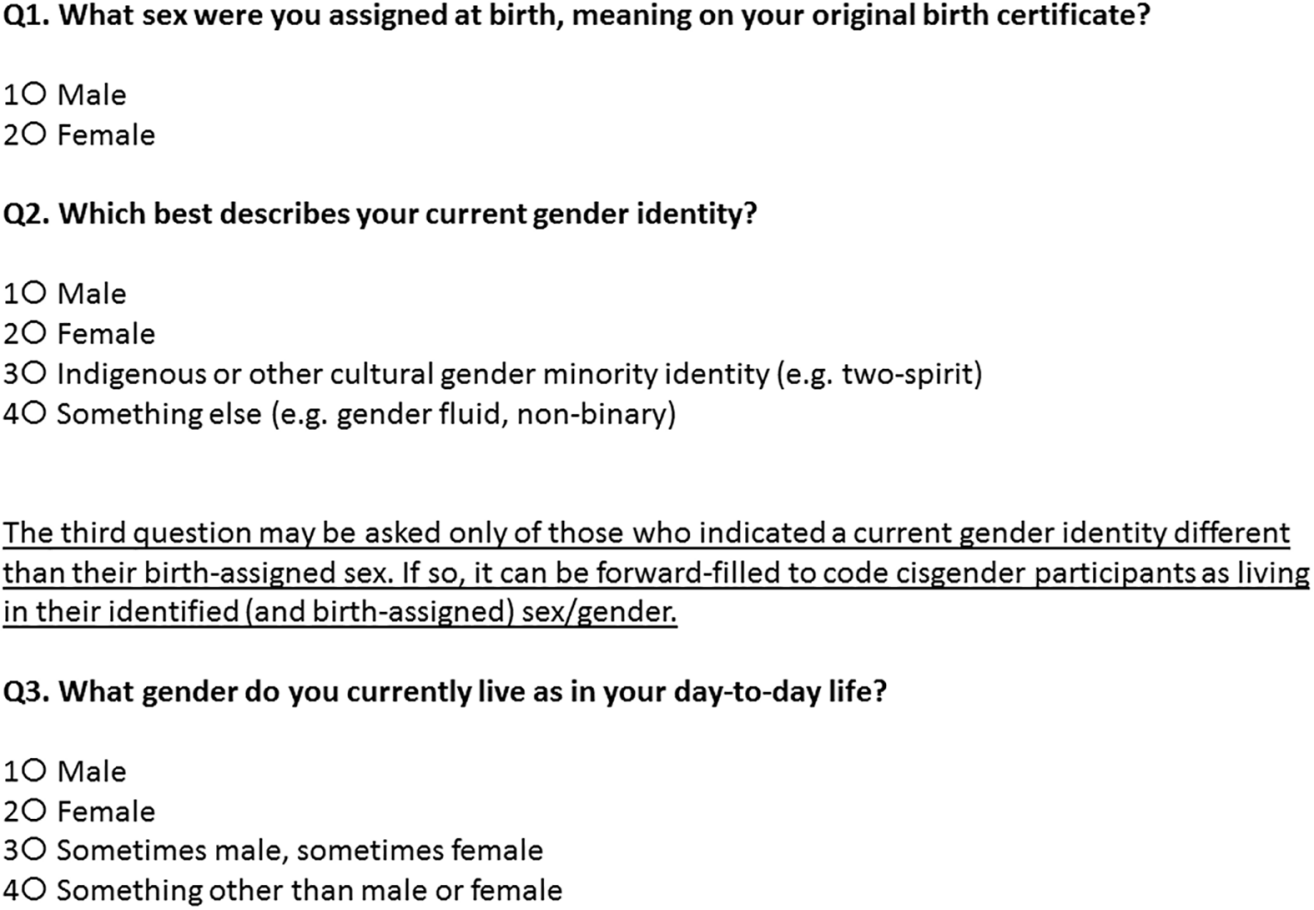
Multidimensional Sex/Gender Measure (MSGM).

For population surveys, we believe that gender identity and lived gender will be the most widely desired sex/gender dimensions, depending on whether health-related analyses are relevant for all those who know themselves to be trans, or for those living in a gender different from that assigned at birth (and thus subject to discrimination and structural barriers such as issues with identity documents). Based on our analysis, and on consultation with a small number of content experts on issues such as population survey design, Indigenous gender concepts, and data uses outside of population health (e.g. human rights), we recommend the following Multidimensional Sex/Gender Measure (MSGM) (Fig 4) for further testing in a Canadian context, and provide recommendations for adaptation in other contexts. Two simple items are required for all participants, and one for a small subset. Optional additions include an open-ended item to capture the complexity of gender identities, and items on sex characteristics (e.g. hormonal treatment) that can be used where relevant for specific health research questions.

These MSGM items should be used in the order provided for telephone or internet surveys, and at least the first two items included so that they can be viewed simultaneously on internet or paper surveys. This question set represents a combination of workable aspects of both item sets tested, and allows for the capture of sex assigned at birth, gender identity and lived gender. By cross-tabulating each of the second and third items with the first, trans-inclusive measures may be created for gender identity and lived gender, that accurately categorize both trans and cisgender respondents. For gender identity, variables can be created with four to eight categories, two of which are cisgender men and women; the remaining categories can differentiate transmasculine and transfeminine persons with Indigenous or cultural gender minority identities, non-binary identities, and male/female identities, or these can be collapsed together for analysis. For lived gender, trans options can be similarly expanded or collapsed according to the needs of the analysis. If only gender identity is desired, then the first two questions alone may be included; if only lived gender is desired, then the first and third questions alone may be included. In general, sex assigned at birth can be cross-tabulated with any other dimension of gender or sex that is relevant to different research questions, to produce a trans-inclusive measure that also differentiates by gender spectrum.

We note that these items are phrased for a self-completed survey, and were tested as such. For interviewer-administered surveys, the items can also be phrased as questions that include the response options, for example “On your original birth certificate, were you assigned a sex of male or female?”

### Rationale and adaptations for Q1

This item is taken from the two-step test measure, and is used in other versions of two-step measures. It performed well in our evaluation. While participants were given an option for undetermined sex at birth on the multidimensional measure’s birth-assigned sex item, an option that is available on Ontario birth certificates, no participant chose this option. While this is not surprising given the sample size, it is unlikely that a participant would check “undetermined” as it is typically given as a short-term provisional classification for infants for whom a sex determination cannot be immediately made. Thus, it is not clear whether participants would even be aware if they had at any point been given an undetermined assignment. We note that if sex/gender markers are removed from birth certificates in the future (something being proposed in some jurisdictions), then the question item stem will need to be adapted as those without an “M” or “F” on their birth certificates become old enough to complete surveys (or have their parents complete, if proxy reporting is used).

### Rationale and adaptations for Q2

This gender identity option makes modifications to both the multidimensional measure and two-step gender identity items to create a new item. First, the question stem asks which option “best describes” the participant’s gender identity. This acknowledges that one’s specific personal identity may not be included, and that researchers are aware this is not a detailed or complete list. Response options do not differentiate between those who personally identify, for example, as men versus trans men, as we found no evidence this was necessary, and these categories are grouped for analysis. While some trans interview participants were clear they were men or women who did not personally identify as trans, none stated that they identified as, for example, a trans man but not a man. We have also added an Indigenous or cultural gender identity response option to recognize the traditional gender identities that exist in many Indigenous cultures within Canada and the world. We included “two-spirit” as an example even though it is a term designed to communicate the concept of Indigenous gender identities to non-Indigenous people, as it is commonly recognized and we were not able to identify any other umbrella term. Finally, we included one response option for other identities such as non-binary, genderqueer, agender, or gender fluid, as participants were not always able to differentiate between options of “both male and female” and “neither male nor female”, and no individual indicated these were particularly important wordings for them.

We designed the third and fourth options to include specific identities as examples rather than in the main response option, in order to allow for changing only these examples as terminology changes over time. Moreover, we avoided including the overly broad term “gender non-conforming”, to reduce the likelihood of misclassifying queer cisgender respondents as trans. We also aimed for wordings and examples that would be clear and easy for interviewers to pronounce, regardless of whether they were part of our cognitive testing. Some options were rejected based on this criterion, e.g. when spoken, “agender” may sound like “a gender” or “agenda”. We believe our suggested response options for Q2 will work well, and that similar to our findings, cisgender participants who are unable to define them will still easily recognize that they do not identify personally with these terms; however, additional testing is required with both Indigenous and non-Indigenous participants.

We note that response options for this second question in particular may need to be adapted for use in other regions. In other contexts, one particular cultural gender identity may be common (e.g. hijra, kathoey, travesti), and can be added as a response option by itself. In other contexts, an Indigenous response option may be unnecessary and can be removed.

### Optional Q2 open-ended follow-up item

We did not include a fill-in “other, please specify” response option for gender identity, as this can require a large amount of time devoted to recoding, and recoding may not reflect participant wishes. Moreover, recoding of some write-ins will not be clear or even possible (e.g. “human”). We believe that asking “which best fits” resolves some concerns in categorization. To provide greater flexibility for participants or to better understand current gender identities, we would suggest adding a separate open-ended question to follow Q2, wherein participants may write in their personal gender identities, rather than adding an “other” option to the item responses. The question stem could be “How do you personally identify your gender?” or something similar. Separating the question allows participants to categorize themselves in Q2, rather than to be categorized by researchers. Those who choose not to include an open-ended question for all respondents may want to consider including one for those who skip Q2.

### Rationale and adaptations for Q3

The lived gender item may be asked only of participants identified as trans by the broader gender identity definition, and this may be programmed into survey skip patterns. This question item is based on the multidimensional measure’s lived gender item, with one small change. While the item tested well, the language of “third gender” is an older term, and not one that any participant personally identified with. Here we just eliminated this term, as it was not needed for clarity. For surveys using proxy reporting, where the reporter will more likely know the lived gender of family or household members than their gender identity, a two-item version consisting of questions 1 and 3 (for all participants) may be a reasonable solution, though question 3 has not yet been evaluated with cisgender participants.

### Optional items on dimensions of sex

Depending on the informational needs regarding sex and/or gender, additional questions on hormone use or surgeries may be included, and in most cases should be asked of cisgender as well as trans participants.

### Coding

SAS syntax for coding is presented in the supplemental materials (S3 File). In addition to the three single variables, this generates four new cross-classified variables that identify trans participants, two for gender identity and two for lived gender. Each dimension has one option that preserves all the categories, and one that collapses to form two categories for cisgender men and women, and trans participants grouped by gender spectrum into transmasculine and transfeminine. Code is also provided for a trans sub-group indicator of living or not living in a gender other than that assigned at birth.

## Conclusions

Both measure sets evaluated performed reasonably well, but with frequent problems identified. Consistent with other research on trans-inclusive survey measures for sex/gender, confusion on the part of cisgender participants was not found to be an issue, indicating little reason for concern with misclassification of this much larger group. In contrast, misclassification of trans respondents will likely be a concern with any single survey item attempting to be inclusive, as a substantial proportion of trans participants will indicate they are simply male or female in line with either their identity, lived gender, or birth-assigned sex. While a single sex/gender question with expanded options beyond male and female may provide greater flexibility for respondents than a male/female question, it will likely similarly fail to adequately identify trans survey respondents. Building on the two measures evaluated and issues identified for measure development, we propose a multidimensional measure of sex and gender for population surveys where data will be used for multiple aims. It includes two base questions on sex assigned at birth and gender identity, and a third item on lived gender asked only of the subset of participants whose gender identity differs from their sex assigned at birth. Additional dimensions of gender or sex (e.g. hormonal milieu) will be relevant for some surveys and can easily be added. Careful specification of sex and gender dimensions assessed, and of response options, can allow for health research which better reflects the diversity of sex and gender characteristics and their relationships to the health of populations.

## Supporting information

S1 File SAS coding for multidimensional test measure. (PDF)

S2 File SAS coding for two-step test measure. (PDF)

S3 File SAS coding for new Multidimensional Sex/Gender Measure. (PDF)
