# Supplementary material for: Transgender-inclusive measures of sex/gender for population surveys: Mixed-methods evaluation and recommendations
Source: PLoS One. 2017 May 25;12(5):e0178043. doi: 10.1371/journal.pone.0178043 (PMC5444783; doi:10.1371/journal.pone.0178043)
Supplement: S1 File — (PDF) [file pone.0178043.s001.pdf]

S1 Appendix. Coding for multidimensional measure.

*NOTE: As A1 contains a fill-in-the-blank option, entries must first be reviewed and recoded if appropriate. Item A6 is check-all-that-apply, so each response option is a separate dichotomous variable taking a value of 1 if checked. Since items A3-A6 are included in a skip pattern, these are first forward-filled so that cisgender individuals (A1 and A2 both male or both female) are recorded as having a consistent sex at birth, gender identity, lived gender, and no transition-related changes to hormones or surgery. Variable names ending in "_ff" designate forward-filled variables.

```
/*-- Multidimensional measure (Bauer, 2012) - SAS syntax for recodes --*/

*Coding categories for "Cross-coded Gender Identity - 1";
if A3_ff=1 then do;
  if A4_ff=1 then MM_ID1='CisM';
  if A4_ff=2 then MM_ID1='MTF';
  if A4_ff in (3,4) then MM_ID1='MTNB';
  if A4_ff=5 then MM_ID1='MTDK';
end;
if A3_ff=2 then do;
  if A4_ff=1 then MM_ID1='FTM';
  if A4_ff=2 then MM_ID1='CisF';
  if A4_ff in (3,4) then MM_ID1='FTNB';
  if A4_ff=5 then MM_ID1='MTDK';
end;

*Coding categories for "Cross-coded Gender Identity - 2";
if MM_ID1='CisF' then MM_ID2='CisF';
  if MM_ID1='CisM' then MM_ID2='CisM';
  if MM_ID1 in ('MTF','MTNB') then MM_ID2='TFEM';
  if MM_ID1 in ('FTM','FTNB') then MM_ID2='TMASC';

*Coding categories for "Cross-coded Lived Gender - 1";
if A3_ff=1 then do;
  if A5_ff=1 then MM_LV1='CisM';
  if A5_ff=2 then MM_LV1='MTF';
  if A5_ff=3 then MM_LV1='MTNB';
  if A5_ff=4 then MM_LV1='MTNB';
end;
if A3_ff=2 then do;
  if A5_ff=1 then MM_LV1='FTM';
  if A5_ff=2 then MM_LV1='CisF';
  if A5_ff=3 then MM_LV1='FTNB';
  if A5_ff=4 then MM_LV1='FTNB';
end;

*Coding categories for "Cross-coded Lived Gender - 2";
if MM_LV1="CisF" then MM_LV2='CisF';
  if MM_LV1="CisM" then MM_LV2='CisM';
  if MM_LV1 in ('MTF','MTNB') then MM_LV2='TFEM';
  if MM_LV1 in ('FTM','FTNB') then MM_LV2='TMASC';

*Coding categories for "Cross-coded Hormonal Sex";
if A3_ff=1 then do;
```

```
  if A6_1_ff=0 then MM_TS='CisM';
  if A6_1_ff=1 then MM_TS='MTF';
end;
if A3_ff=2 then do;
  if A6_1_ff=0 then MM_TS='CisF';
  if A6_1_ff=1 then MM_TS='FTM';
end;
```
