# Supplementary material for: Transgender-inclusive measures of sex/gender for population surveys: Mixed-methods evaluation and recommendations
Source: PLoS One. 2017 May 25;12(5):e0178043. doi: 10.1371/journal.pone.0178043 (PMC5444783; doi:10.1371/journal.pone.0178043)
Supplement: S2 File — (PDF) [file pone.0178043.s002.pdf]

S2 Appendix: Coding for two-step measure.

*NOTE: First, B1 is hand-recoded to B1_rec, which incorporates write-in measures.

```
/*-- Two-step test measure (GenIUSS Group, 2014)– SAS syntax for recodes --*/

*Creating categories for "Cross-coded Gender Identity – 1";
if B2=1 then do;
  if B1_rec=1 then TWOSTEP1='CisM';
  if B1_rec=2 then TWOSTEP1='MTF';
  if B1_rec=3 then TWOSTEP1='error';
  if B1_rec=4 then TWOSTEP1='MTF';
  if B1_rec=5 then TWOSTEP1='MTNB';
end;
if B2=2 then do;
  if B1_rec=1 then TWOSTEP1='FTM';
  if B1_rec=2 then TWOSTEP1='CisF';
  if B1_rec=3 then TWOSTEP1='FTM';
  if B1_rec=4 then TWOSTEP1='error';
  if B1_rec=5 then TWOSTEP1='FTNB';
end;

*Creating categories for "Cross-coded Gender Identity – 2";
if TWOSTEP1='CisF' then TWOSTEP2='CisF';
  if TWOSTEP1='CisM' then TWOSTEP2='CisM';
  if TWOSTEP1 in ('MTF','MTNB') then TWOSTEP2='TFEM';
  if TWOSTEP1 in ('FTM','FTNB') then TWOSTEP2='TMASC';
```
