# Supplementary material for: Transgender-inclusive measures of sex/gender for population surveys: Mixed-methods evaluation and recommendations
Source: PLoS One. 2017 May 25;12(5):e0178043. doi: 10.1371/journal.pone.0178043 (PMC5444783; doi:10.1371/journal.pone.0178043)
Supplement: S3 File — (PDF) [file pone.0178043.s003.pdf]

S3 Appendix: Coding for new multidimensional sex/gender measure.

*NOTE: No write-ins, so no hand-recoding required. If variable Q3 is asked only of those who are not cisgender based on Q1 and Q2, then Q3 must be forward filled to indicate a male or female lived gender for cisgender participants. The coding below reflect this in that Q3_ff indicates a forward-filled variable.

```
/*-- New Multidimensional Sex/Gender Measure – SAS syntax for recodes --*/

*Creating categories for “Cross-coded Gender Identity – 1”;
if Q1=1 then do;
  if Q2=1 then MSGM_ID1='CisM';
  if Q2=2 then MSGM_ID1='MTF';
  if Q2=3 then MSGM_ID1='MT2S';
  if Q2=4 then MSGM_ID1='MTNB';
end;
if Q1=2 then do;
  if Q2=1 then MSGM_ID1='FTM';
  if Q2=2 then MSGM_ID1='CisF';
  if Q2=3 then MSGM_ID1='FT2S';
  if Q2=4 then MSGM_ID1='FTNB';
end;

*Creating categories for “Cross-coded Gender Identity – 2”;
if MSGM_ID1=’CisF’ then MSGM_ID2='CisF';
  if MSGM_ID1=’CisM’ then MSGM_ID2='CisM';
  if MSGM_ID1 in ('MTF',’MT2S’,'MTNB') then MSGM_ID2='TFEM';
  if MSGM_ID1 in ('FTM',’FT2S’,'FTNB') then MSGM_ID2='TMASC';

*Coding categories for “Cross-coded Lived Gender – 1”;
if Q1=1 then do;
  if Q3_ff=1 then MSGM_LV1='CisM';
  if Q3_ff=2 then MSGM_LV1='MTF';
  if Q3_ff in (3,4) then MSGM_LV1='MTNB';
end;
if Q1=2 then do;
  if Q3_ff=1 then MSGM_LV1='FTM';
  if Q3_ff=2 then MSGM_LV1='CisF';
  if Q3_ff in (3,4) then MSGM_LV1='FTNB';
end;

*Coding categories for “Cross-coded Lived Gender – 2”;
if MSGM_LV1="CisF" then MSGM_LV2='CisF';
  if MSGM_LV1="CisM" then MSGM_LV2='CisM';
  if MSGM_LV1 in ('MTF','MTNB') then MSGM_LV2='TFEM';
  if MSGM_LV1 in ('FTM','FTNB') then MSGM_LV2='TMASC';

*If desired, coding for whether living in gender one identifies as;
*For trans subgroup;
if MSGM_ID2 in (‘TFEM’,’TMASC’) then MSGM_OUT=0;
  if MSGM_ID2=’TFEM’ and MSGM_LV2=’TFEM’ then MSGM_OUT=1;
  if MSGM_ID2=’TMASC’ and MSGM_LV2=’TMASC’ then MSGM_OUT=1;
```
